# Research on the Mixed Education Mode for the Safety Engineering Major during the Coronavirus (COVID-19) Epidemic

**DOI:** 10.3390/ijerph19041967

**Published:** 2022-02-10

**Authors:** Kai Yu, Lirong Wu, Lujie Zhou

**Affiliations:** 1 College of Mining and Safety Engineering, Shandong University of Science and Technology, Qingdao 266590, China; lrwu1981@163.com (L.W.); zhouqingdao@yeah.net (L.Z.); 2 Min an Institute of Emergency and Safety Management of Qingdao West Coast New Area, Qingdao 266590, China

**Keywords:** higher education, education model, online, offline, COVID-19

## Abstract

During the COVID-19 epidemic, many countries faced a critical situation in terms of the global economy and human social activities, including education. In China, the coronavirus is better controlled. Chinese university students have returned to school to study. Despite previous research on online education and learning, the readiness of students for the online and offline learning models implemented at this particular time is not well understood. This paper discusses a hybrid education model for undergraduate students in the safety engineering major. Questionnaires are administered to faculty and students from different colleges and universities in the same major to statistically summarize the influencing factors of mixed or hybrid education. The system dynamics (SD) model is constructed and simulated to determine that using online in the tenth to fifteenth, twenty-fifth to thirtieth, and fortieth to forty-fifth min of classroom teaching (50 min in total) can effectively increase students’ interest and engagement in learning. More hands-on activities should also be considered to enhance students’ motivation to acquire knowledge, and consideration could be given to encourage interaction among students. This study will be continuously improved by a follow-up study of undergraduate student performance. This study has important implications for educators implementing online and offline blended instruction.

## 1. Introduction

The COVID-19 epidemic spread rapidly in China in early 2020. The number of people with the disease continued to increase globally until 2022. Despite the downward trend of new cases in China, the virus is still spreading in a very small area in China. COVID-19 is hampering the global economy [1] and affecting human social activities, especially education. During the outbreak of the epidemic, many countries and regions were forced to suspend face-to-face teaching in the classroom [2,3].

In response to the COVID-19 outbreak, online learning seemed to be the only solution for the education sector. While offline classes have been discontinued, institutions of higher learning around the world have revisited the feasibility of online learning to minimize the impact on student academics [4,5]. Online teaching and learning allow educational activities to continue and minimize the impact on students’ learning progress. However, there are still some challenges in implementing online learning [6,7].

The advantages and benefits of blended learning approaches to optimize teaching and learning are evident in numerous influential studies and are considered by many scholars to be the “new normal” in education [8]. The current challenges of blended learning are revealed from the perspective of students, teachers, and institutions [9]. Blended learning increases interaction between instructors and students. It also offers flexibility, rich content, and improved cost-effectiveness [10,11]. In actual online teaching studies, it has been found that it requires higher levels of basic computer skills, self-control, human-computer interaction, and motivation to learn compared to classroom learning [12,13,14,15]. Student-teacher and student-student interactions contribute more to instructional effectiveness than student-content interactions [16]. The type of interaction between learning group participants was a key influence in its formation [17]. Originally geared toward informal learning, MOOCs have recently begun to be embraced as part of formal campus education [18].

Several studies have reported problems with the components of blended instruction, on the part of students [19,20], teachers, and educational institutions [21,22,23]. Some studies also feature a single type of blended learning report. The research on the advantages and challenges of the flipped classroom was limited to this type of blended learning and reported specifically on the technical challenges found in the flipped classroom [24]. Brown examined the challenges from the teacher’s perspective [25]. The study found that teachers’ technology anxiety, complexity, and students’ lack of technology were the challenges they encountered when using online technologies for teaching.

In addition, some of the most notable recent blended learning research has focused on overall design challenges, rather than focusing specifically on the online component. The study by Boelens et al. identified “merging flexibility”, “facilitating interaction”, “facilitating the learning process of students”, and “fostering an effective learning climate” as “four key challenges for blended design” [26]. Similarly, Graham and his team provided such a contribution by providing a framework, direction, and guidelines for educational institutions and also considered blended learning (face-to-face and online components) as a whole [27,28,29].

During the epidemic period, our teaching team has accumulated rich experience in online teaching and found out its advantages. The epidemic in China has been effectively controlled, enabling college students to return to the classroom to continue their studies. The research questions how to introduce online teaching into classroom teaching to improve the overall teaching effect. This provides a good opportunity to study online and offline blended learning. Based on the classroom teaching of safety system engineering, this paper will determine the best combination of online courses by simulation method to improve the teaching effect.

## 2. The State of the Art

Many countries have implemented a variety of policies to control the situation, including border controls and public health policies. These measures have also affected the education sector, forcing the closure of schools in many countries [30,31]. During COVID-19 outbreaks, many countries suspended face-to-face classroom instruction from the early stages of the outbreak [32]. Higher education was forced to use online teaching technologies to solve the problem of teaching during the COVID-19 epidemic.

During the outbreak of the epidemic, blended teaching was the main teaching method of higher education. In blended learning, learning outcomes were positively correlated with the depth of online technology and the duration of interaction with online summative tasks [33]. Students faced higher challenges than usual in establishing mixed learning habits. During the COVID-19 pandemic, the learning environment helped them develop learning habits to play a greater role [34]. Compared to the past, in blended learning, students are more likely to accept e-learning methods [35]. The mixed teaching mode is superior to the traditional teaching mode in enriching students’ professional knowledge and cultivating students’ comprehensive ability. It can effectively improve the quality of education, improve students’ learning effect, and enhance students’ satisfaction [36,37,38].

When instructors use interactive technology consistently and purposefully, students feel more connected to the instructor and their peers in an online course [39]. It is worth stating that the sudden shift in campus education from traditional face-to-face education to an online learning environment has also created challenges for higher education. During the COVID-19 epidemic, students’ emotional input and interpersonal communication level (student-student or student-teacher) have decreased after experiencing the transformation from traditional to online learning environment [40]. On the student side, in some areas of China, a small number of students do not have Internet access, have slow Internet connections at home, or need to bypass firewalls [41,42]. Nonetheless, online teaching has been continuously adapted and improved; it has contributed greatly to Chinese higher education during the COVID-19 epidemic. Therefore, based on the previous research on online education, this paper will continue to study in-depth the hybrid online and offline model of higher education.

To improve teaching effectiveness, numerous experts are simultaneously trying to use simulation to study higher education. Simulation research in medical education has been explored in-depth in terms of models and techniques [43,44,45]. Fishbone diagrams can thoroughly analyze the constituent elements of a problem and, therefore, are often used to analyze the influencing factors of a problem [46]. System dynamics (SD) is capable of qualitatively analyzing complex dynamic processes with multiple factors and is often used in simulations to analyze the change process of complex problems [47].

The methods, technologies, and challenges of online teaching have been widely studied. The mixed education model of higher education has also received attention during the COVID-19 epidemic. The combination of online and offline teaching methods in the mixed teaching mode and its research methods still need to be deeply studied.

In order not to disturb undergraduate students’ learning as much as possible, actual teaching cannot be easily used as a test subject. Therefore, this paper proposes to use the simulation method to analyze the optimal combination mode of online and offline teaching, and to study the problem according to this research question. The simulation method of fishbone diagram and system dynamics was used in this study. A new combination of online and offline teaching is identified and analyzed by simulations. This then guides the actual teaching during the COVID-19 epidemic.

## 3. Methods

### 3.1. Influencing Factors

**Figure 1:**
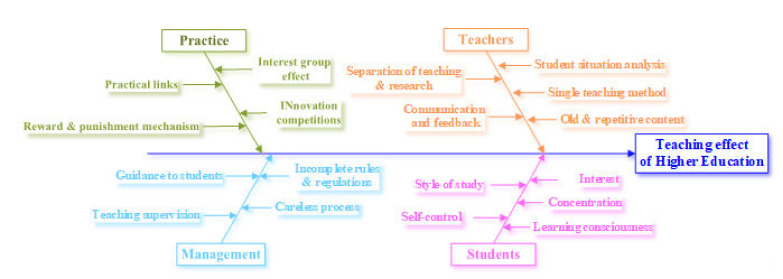
Analysis of influencing factors of higher education teaching effect.

To fundamentally investigate the factors influencing the effectiveness of higher education teaching, this study uses “higher education teaching effectiveness” as the outcome and uses fishbone diagrams to analyze the causes of this outcome. Practice, management, teachers, and students are taken as the main causes. The medium causes, that cause each main cause, and the minor causes, that cause the medium causes, are analyzed until measures can be taken [48,49], as shown in Figure 1.

In the teaching process of higher education, teachers and students are the main body of activities. Teachers are the foundation of education, the source of promoting education, and the output of knowledge. Teachers’ values, teaching methods, and teaching contents directly affect the effect of teaching. Students are the input of knowledge. Their attention, self-control, and interest are the key factors affecting the learning effect, which will affect other students. The factors affecting students’ practical ability mainly include practical innovation activities and extracurricular activities. In addition, it benefits from comprehensive and systematic policies, systems, effective management, and supervision that excellent teaching teams, a good learning atmosphere, effective practical activities, and extracurricular activities are able to provide.

### 3.2. Investigation and Research

The questionnaire is designed according to the characteristics of the influencing factors of the higher education teaching effect. The questionnaire is divided into four parts: completing instructions, survey purpose and introduction to relevant concepts, basic personal information of respondents, and a survey of higher education teaching information. The option design of related factors adopts Likert-type scale to improve the accuracy of quantitative analysis. The respondents selected the options of each test item, and the attribute values of the five options were scored from low to high, with 1–5 respectively.

The research objects mainly include undergraduates and teachers in colleges and universities. A total of 400 questionnaires were distributed and 378 were recovered, with a recovery rate of 94.50%. Incomplete questionnaires (such as missing questions) and invalid questionnaires that do not meet the requirements are eliminated. There are 367 valid questionnaires, and the effective recovery rate is 91.75%.

**Figure 2:**
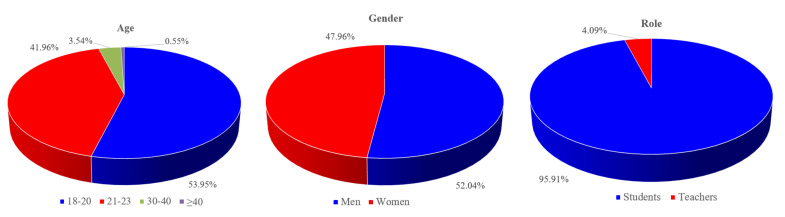
Distribution of age, gender, and role.

Figure 2 shows the statistical information of the basic information of the questionnaire survey. It can be seen that the sample structure of this survey is reasonable, and the statistical information of respondents’ age, gender, and role is consistent with the actual situation of colleges and universities, which effectively ensures the objectivity of the survey results.

Cronbach (α) is used to analyze the reliability of the questionnaire. The α is calculated to be 0.841, which is consistent with 0.7 < α < 0.9, indicating that the questionnaire results are highly reliable. Validity refers to the extent to which the questionnaire can correctly measure the investigated factors. Criterion-related validity analysis was used. If the correlation is significant, it is an effective item. The calculation shows that each item reaches the significant level of 0.01, indicating that the correlation degree of calibration is good.

### 3.3. Model

Based on the analysis of influencing factors and the questionnaire, the SD model of higher education effect is constructed. The SD model is simulated and analyzed to explore the best way of combining online and offline higher education.

The multiple linear regression coefficients of the questionnaire were analyzed. Taking variable Attention as an example, the regression coefficient is shown in Table 1.

Then, the Attention can be expressed as Equation (1) in SD model.

*A* = 0.781 × *s* + 0.516 × *l* + 0.423 × *c* + *b* (1)

where, *A* is attention, *s* is students’ style of study, *l* is learning interest, *c* is students’ self-control, and *b* is a constant, its value in this paper is 0.004.

Other relations of the SD model are as follows.

*T* = *b_t_*_1_ × *m* × *o* + 0.892 × *p* + 0.513 × *r* + 0.573 × *l_c_* + 0.691 × *w* + 0.125 × *t_r_ + b_t_*_2_ (2)

where, *t* is teacher teaching, *m* is efficiency of mixed mode, *o* is online teaching, *p* is practice, *r* is rules and regulations, *l_c_* is lecture content, *w* is way of teaching, *t_r_* is teaching and scientific research, *b*_t1_ and *b_t_*_2_ are constants, their values in this paper are 0.021 and 0.009.

*M* = 0.319 × *c_i_* + 0.214 × *a_s_* + *b_m_* (3)

where, *c_i_* is continuous improvement, *a_s_* is analysis of academic situation, *b_m_* is constant, its value is 0.015.

a_s_ = *b_as_*_1_ × (0.971 × *a_l_* − *T_e_*)/0.259 × *a* (4)

where, *a_l_* is awareness of learning, *T_e_* is teaching effect of higher education, *b_as_*_1_ is constant, its value is 0.011.

*P* = 0.693 × *i_g_* + 0.591 × *i_a_* + 0.317 × *l_d_* + *b_p_* (5)

where, *i_g_* is interest group, *i_a_* is innovation activities, *l_d_* is link design, *b_p_* is constant, its value is 0.008.

*o* = 0.989 × *t_s_* + 0.391 × *p_t_* + *b_o_* (6)

where, *t_s_* is teaching supervision, *p_t_* is process teaching, *b_o_* is constant, its value is 0.025.

*a*_l_ = *b*_*al*1_ × *c_i_* × *g_s_* + *b*_*al*2_ (7)

where, *g_s_* is guidance to students, *b_al_*_1_ and *b_al_*_2_ are constants, their values are 0.029 and 0.008.

*c_t_* = [1 + (−1)*^sgn^*^(*λct*)^*λc_t_*] × INTEG(*t_i_*) = INTEG[*b_ct_*_1_ × (*t* − *c_t_*)/*s_s_* + 0.133 × *p_t_* + 0.145 × *t_s_ + b_ct_*_2_]

λ*c*_*t*_ = (*c*_*end*_ − *c*_0_)/*c*_0_

(8) $$\mathrm{sgn}\lambda c_{t}=\left\{\begin{array}{l} 0\;\;\;\;\;c_{t}<\mu \\ 1\;\;\;\;\;c_{t}\geq \mu \end{array}\right.$$

where, *c_t_* is classroom teaching, *t_i_* is teaching increment, *s_s_* is students’ self-control, *b_ct_*_1_ and *b_ct_*_2_ are constants, their values are 0.017 and 0.006. *c_end_* is the final result of the simulation, *c*_0_ is the initial value of the simulation (*c_end_* and *c*_0_ are usually the initial values and results of the last same simulation).

*T_e_* = [1 + (−1)*^sgn^*^(*λTe*)^*λT_e_*] × INTEG(0.897 × *e_i_* + 0.469 × *c_i_ + b_Te_*) = INTEG(0.897 × *o* × *c_t_* + 0.469 × *c_i_ + b_Te_*)

λ*T*_*e*_ = (*T*_*end*_ − *T*_0_)/T_0_

(9) $$\mathrm{sgn}\lambda T_{e}=\left\{\begin{array}{l} 0\;\;\;\;\;T_{e}<\mu \\ 1\;\;\;\;\;T_{e}\geq \mu \end{array}\right.$$

where, *T_e_* is teaching effect of higher education, *e_i_* is effect increment, *b_Te_* is constant, its value is 0.004, *T_end_* is the final result of the simulation, *T*_0_ is the initial value of the simulation (*T_end_* and *T*_0_ are usually the initial values and results of the last same simulation).

The variables *l*, *c*, *o,* and *a_l_* in the model are functions of sigmoid of *time*, as Equations (10)–(13).

*L* = sigmoid (*time*) = 1/(1 + e^−*time*^) + *c_intl_* (10)

where, *c_intl_* is the initial value of *l*.

*C* = sigmoid (*time*) = 1/(1 + e^−*time*^) + *c_intc_* (11)

where, *c_intc_* is the initial value of *c*.

*O* = sigmoid (*time*) = 1/(1 + e^−*time*^) + *c_into_* (12)

where, *c_into_* is the initial value of *o*.

*a_l_* = sigmoid (*time*) = 1/(1 + e^−*time*^) + *c_intal_* (13)

where, *c_intal_* is the initial value of *a_l_*.

**Figure 3:**
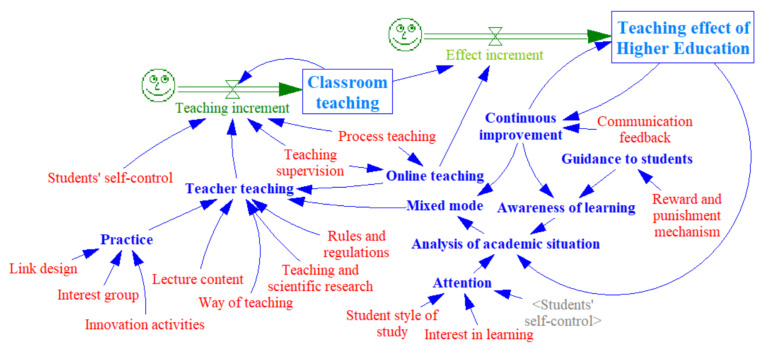
SD model of Higher Education.

The SD model of the higher education teaching effect is constructed according to this method, as shown in Figure 3.

## 4. Results

The initial state is used as a reference to compare and analyze the impact of different simulation strategies on the effect of hybrid teaching.

### 4.1. Initial State

**Figure 4:**
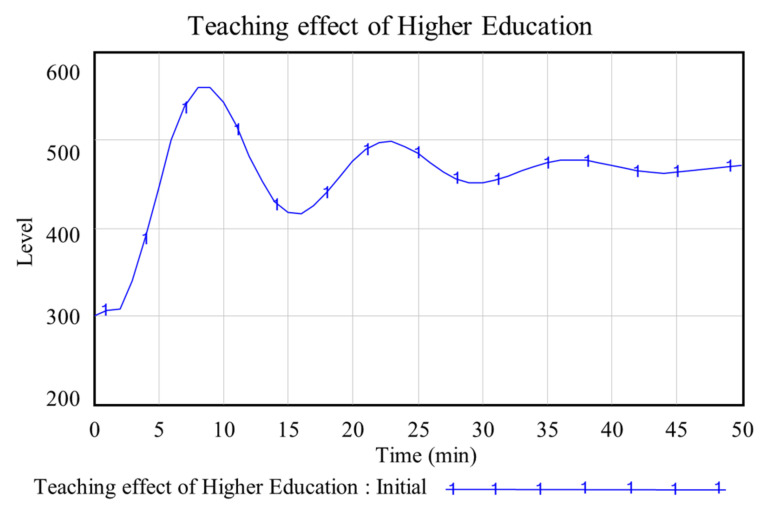
Trend of higher education teaching effect in the initial state.

The simulation time is set to 50 min because Chinese universities usually have 50 min per class. The simulation step is set to one minute. In the initial state, the teaching effect of higher education is shown in Table 2, and the specific change trend is shown in Figure 4.

Combined with Table 2, Figure 4 showed that the teaching effect reached its peak in the ninth min (559.25). This was because, from the beginning of class, teachers quickly adjusted the teaching state, and students’ attention was gradually focused. Then, the teaching effect decreased gradually. At the sixteenth min, the teaching effect decreased to 415.91. This is because after time, students gradually relaxed and became distracted in class. Subsequently, the teaching effect fluctuated. At the twenty-third min, it rose again to 497.71. The main reason was that students adjusted their listening state in time. However, this was limited to some students with good grades. This also explained that the teaching effect will rise, but it will not reach the state of the ninth minute. Until the end of class (the fiftieth min), the teaching effect fluctuated.

The effect at the end of class (the fiftieth min) increased by 56.94%, compared with the teaching effect at the beginning of class (zero min). After class, the effect decreased by 15.81%, compared with the peak of the teaching effect (the ninth min).

### 4.2. Strategies Simulation

**Figure 5:**
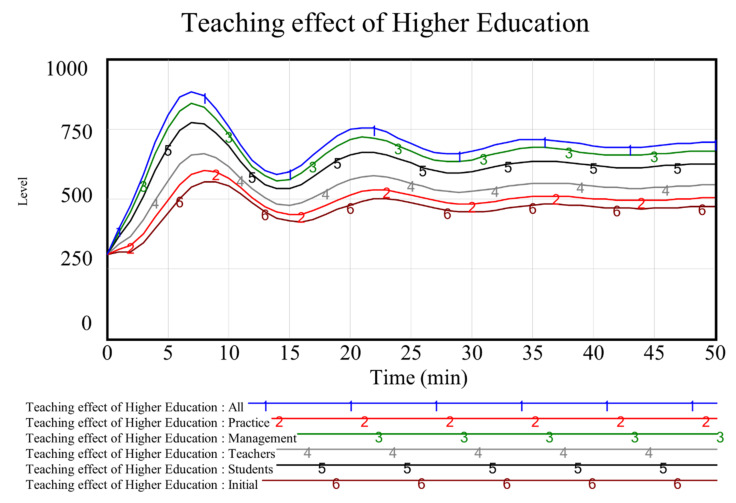
Teaching effect trend of higher education with different strategies.

According to the analysis of the influencing factors of higher education teaching effect (Figure 1), this study explores and analyses better improvement methods of education and the teaching effect from the aspects of improving the measured intensity of teachers, students, management, and practice. The trend of different strategy simulation of the higher education teaching effect is shown in Figure 5.

It can be seen from the changing trend in Figure 5 that the teaching effect is the best way to enhance the effect of all measures (Line 1). This is well known and also verifies the reliability of the simulation. When changing single measures, the teaching effect from high to low is management, students, teachers, and practice. Compared with the initial state, the degree of change is shown in Table 3.

As can be seen from Table 3, if the measures in one aspect are strengthened, the time required for the teaching effect to reach the peak will be reduced. The time of management, students, teachers, and practice is advanced by 22.22%, 22.22%, 11.11%, and 11.11% respectively, compared with the initial state. At the end of class, the teaching effect is improved by 42.04%, 16.17%, 32.34%, and 6.47% respectively.

It can be seen from Figure 5 and Table 3 that the teaching effect always fluctuates whether one measure or all measures are enhanced. This is closely related to the change in students’ attention. To verify the assumptions, this study further explores the relationship between attention and the teaching effect to find a better opportunity for the combination of online and offline teaching.

**Figure 6:**
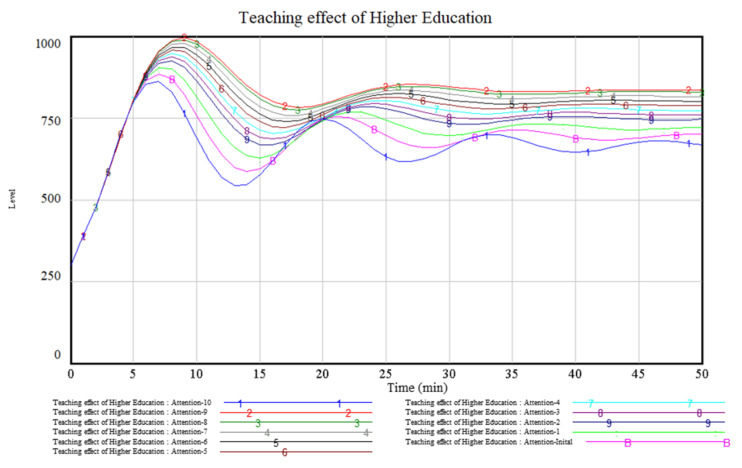
Attention sensitivity analysis.

This study analyses the sensitivity of attention to the teaching effect, as shown in Figure 6. It can be seen from Figure 6 that the teaching effect is significantly improved by improving students’ attention in class. The highest value of the teaching effect increased from 867.33 to 995.15, an increase of 14.74%. After class, the effect also increased from 699.19 to 835.13, an increase of 0.17%. Moreover, with the improvement of attention, the fluctuation range of the teaching effect decreases gradually.

However, a careful analysis of Figure 6 reveals that it is not the case that higher attention is better. When attention is raised to a certain level and continues to be raised, the teaching effectiveness decreases instead. The reason is that with a high level of concentration, students tend to feel tired, which in turn will affect their learning.

## 5. Discussion

### 5.1. Analysis of Results

Figure 5 shows that the teaching effect is poor in the tenth to fifteenth, twenty-fifth to thirtieth, and fortieth to forty-fifth min. The simulation analysis in Figure 6 shows that focusing students’ attention can effectively improve teaching effectiveness. However, how and when to improve students’ attention is the focus of this paper’s in-depth investigation in actual teaching.

The simulation shows that the tenth to fifteenth, twenty-fifth to thirtieth, and fortieth to forty-fifth min are the key periods to improve the teaching effect. During the above-mentioned period, this study will promote students’ attention with the help of online teaching methods in classroom teaching.

### 5.2. Application Analysis

The methods used in classroom teaching are as follows.

Show, Examine, and Help (SEH): The classroom atmosphere is enhanced; theoretical knowledge is consolidated; and students’ ability to think, summarize, and communicate is cultivated by student-student interaction and teacher-student interaction.

Debate: The consolidation of knowledge and thinking are enhanced. Critical dialectical thinking is cultivated.

Flipped Classroom: Cultivate students’ comprehensive application ability and innovation ability.

The online activities used in the above method are shown in Table 4.

Online courses are rich in content with a high frequency of use by teachers and a high degree of participation by students, which has played a positive role in improving the teaching effect.

In addition, in the process of online and offline teaching, we pay attention to cultivating students’ practical cognition. For example, students entering the laboratory for on-site learning to understand the situation of enterprise safety production management and their firefighting emergency drills.

Students’ understanding of theoretical knowledge learned in class is enhanced, the on-site situation of safety production is understood, and their emergency response ability is improved by offline practical activities.

### 5.3. Effect of Practical Teaching

The evaluation of the learning effect is not only the key to measuring students’ actual performance, but is also the key to testing teaching practice and students’ learning effectiveness [50,51].

This study also constructs an online and offline mixed teaching effect evaluation and continuous improvement model. The evaluation of the effect of online and offline mixed teaching is mainly through the assessment of students’ usual performance and final examination results. Different evaluation objectives have different weights. The ratio of the average score of all students in each goal to the preset total score of the goal indicates the completion of students. The overall achievement is represented by the execution weighting of all objectives. If the result is more than 60% (0.6), it means that the teaching effect is qualified; otherwise, it is unqualified. The evaluation algorithm [52] of this study is shown in Equation (14).

(14) $$E=\left\{\begin{array}{l} 1,G_{s}\geq 0.6\\ 0,G_{s}<0.6 \end{array}\right.\;\;G_{s}=\sum_{i=1}^{n}\frac{Ta_{i}\;}{Tt_{i}}\times W_{i}$$

where, *E* is the completion of the target (1 means the goal is reached, and 0 means the goal is not reached), *G_s_* is the evaluation value of the *s*-*th* course objective, *i* is the assessment method of the course (online assessment, offline assessment, homework, etc.), *Ta_i_* is the average score of assessment method *i*, *Tt_i_* is the total score of assessment method *i*, and *W_i_* is the weight of assessment method *i*.

**Figure 7:**
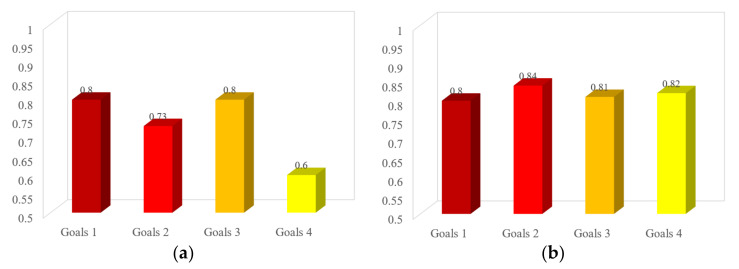

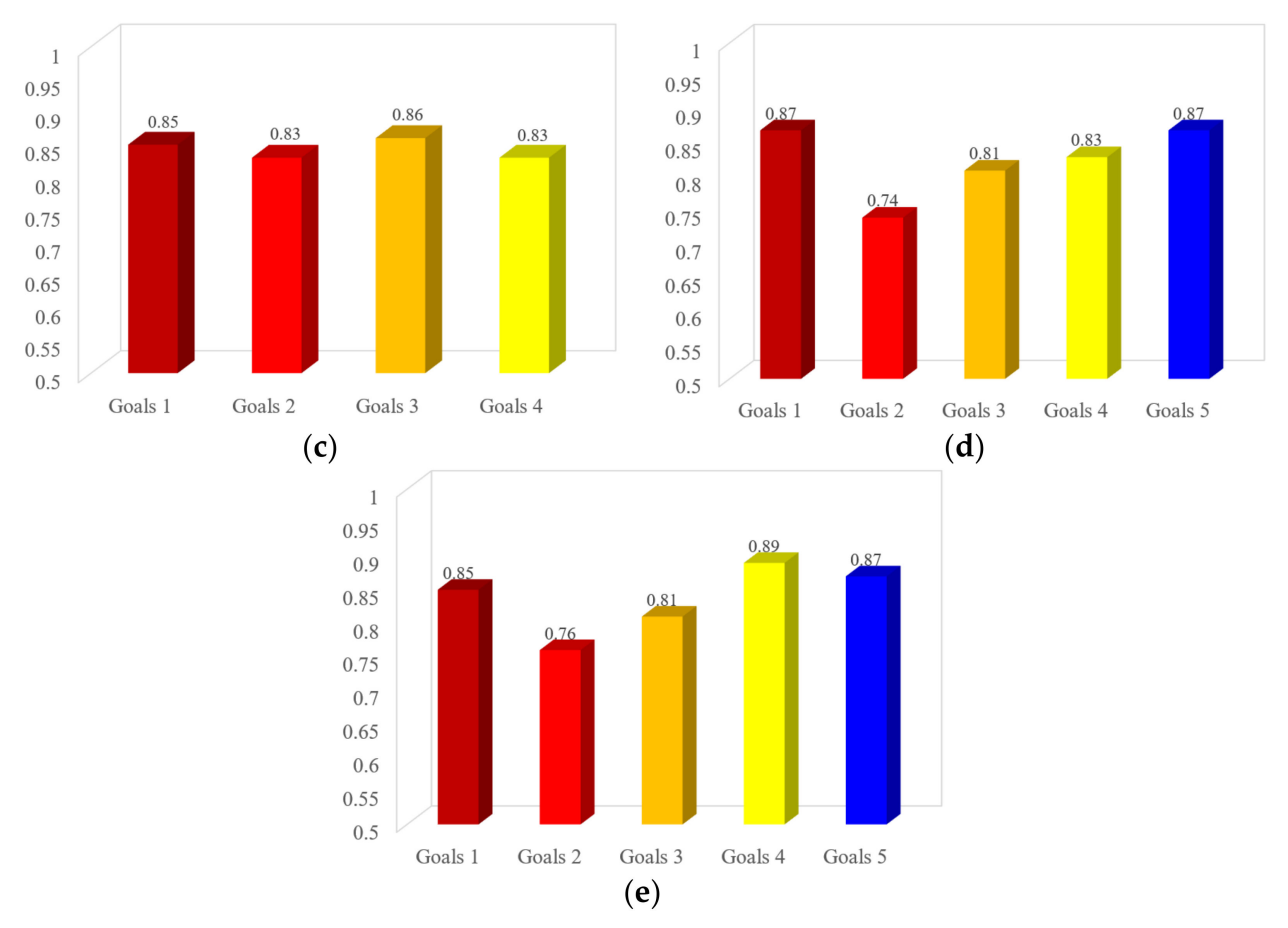
Analysis of the teaching effect. (**a**) The 1st teaching cycle of safety evaluation. (**b**) The 2nd teaching cycle of safety evaluation. (**c**) The 3rd teaching cycle of safety evaluation. (**d**) The 1st teaching cycle of safety system engineering. (**e**) The 2nd teaching cycle of safety system engineering.

Figure 7 shows the analysis of the mixed education effect. Figure 7a,b are the teaching effect analysis of safety evaluation in three teaching cycles (professional courses), and Figure 7d,e are that of safety system engineering in two teaching cycles (professional courses). The analysis of two courses (safety evaluation and safety system engineering) (Figure 7) shows that the effect of online and offline mixed education is obvious. After strengthening all-around measures and using online teaching methods to promote students’ attention, the teaching effect has been significantly improved.

However, Figure 7 shows that there is still room for improvement in the teaching effect. In every teaching cycle, the teaching goals fluctuate. Every teaching goal of safety evaluation can be more than 0.8, and that of safety system engineering can be more than 0.7. This will be the direction of continuous improvement of our teaching team. On this basis, we will continue to deeply study teaching theories and methods to better and comprehensively improve the teaching level of the safety engineering specialty in colleges and universities.

## 6. Conclusions

This paper studies the online and offline mixed education mode for undergraduates majoring in safety engineering at colleges and universities. A fishbone diagram is used to construct the influencing factor system of the mixed education model. Then, the questionnaire survey method is used to investigate the teachers and students on safety engineering specialty. On this basis, SD simulation is carried out. The new online and offline hybrid education model is proposed according to the simulation results.

It is found that adding online teaching activities in the tenth to fifteenth, twenty-fifth to thirtieth, and fortieth to forty-fifth of classroom teaching can effectively promote students’ attention, interest, and participation to improve the teaching effect. This study also focuses on strengthening the cultivation of students’ practical abilities. The methods of online and offline mixed teaching evaluation and continuous improvement are proposed. In addition, the teaching cycles practical application of two professional courses verifies the feasibility and reliability of this study. This study provides a theoretical analysis method and practical application system for the cultivation of students majoring in safety engineering in colleges and universities.

However, this study is limited to the teaching of safety engineering. The teaching methods of different courses in different disciplines will be different. Therefore, the research in this paper may not be applicable to higher education in all disciplines. The research method of this paper can provide method reference for higher education of other disciplines. In future research, we will pay more attention to the research of higher education methods and simulation analysis methods.

## Figures and Tables

**Table 1:** Coefficient analysis of variable Attention.

Influence Factors		Regression Coefficient Analysis					
		Coefficient	Standard Deviation	*T*	*P_t_*	*F*	*P_F_*
	(Constant)	0.004	0.142				
	Style of study	0.781	0.038	2.493	0.021	2.495	0.013
	Interest in learning	0.516	0.035	1.015	0.011	2.369	0.015
	Self-control	0.423	0.035	1.311	0.017	1.332	0.016

**Table 2:** Numerical changes of higher education teaching effect in the initial state.

Time (min)	Teaching Effect of Higher Education	Time (min)	Teaching Effect of Higher Education
0	300	26	474.0615234
1	306.6666565	27	464.0975342
2	308.333313	28	456.3323669
3	339.3209839	29	451.8782654
4	389.5473328	30	451.069519
5	446.8610229	31	453.5158691
6	499.5984192	32	458.2820435
7	538.6713257	33	464.1417236
8	558.8683472	34	469.8478088
9	559.2457275	35	474.3663635
10	542.6698608	36	477.0365601
11	514.7219238	37	477.6377258
12	482.262207	38	476.3650513
13	451.9701843	39	473.731842
14	429.1343384	40	470.4273071
15	416.8789368	41	467.1628418
16	415.9060059	42	464.5370178
17	424.7242432	43	462.9414978
18	440.2501221	44	462.519104
19	458.6147461	45	463.1742554
20	475.9966736	46	464.6260071
21	489.3226318	47	466.4874878
22	496.7257385	48	468.353302
23	497.7125244	49	469.8772583
24	493.0507813	50	470.8278198
25	484.4406433		

**Table 3:** Changes in teaching effects with different strategies.

Order	Strategies	Highest Value		After Class (the 50th min)
		Effect	Time	
1	Management	49.86%	−22.22%	42.04%
2	Students	37.50%	−22.22%	16.17%
3	Teachers	18.32%	−11.11%	32.34%
4	Practice	7.29%	−11.11%	6.47%
5	All	58.09%	−22.22%	48.50%

**Table 4:** Activity data statistics of online courses.

Category	Content	Data
Selected courses	Number of selected courses	342
Available resources for the course	Teaching video	84
	Total teaching video time (min)	1000
	Non video resources	10
Announcement	Course announcement	386
Activity	Total number of distribution activities	167
	Total number of participants	6614
	Total number of sign-ins issued	116
	Total attendance	5544
	Total number of votes cast	72
	Total number of questionnaires issued	15
	Total number of participation questionnaires	304
	Total number of responses	365
	Total number of participating scores	78
	Total number of in-class exercises	36
	Total number of tasks involved in grouping	203
Tests and assignments	Total times	161
	Total number of exercises	410
	Number of participants	339
Interactive communication	Total posts	4968
	Number of teacher posts	300
	Number of participants	300
Examine	Times	10
	Total number of test questions	184
	Number of participants	217

## Data Availability

The data presented in this study are available on request from the corresponding author.
